# Medical News and Items

**Published:** 1876-11

**Authors:** 


### Medical News and Items.

A Veteran.—Prof. V. Sigmund, of the University of Vienna, records the case of an old soldier, dying at the age of eigthy-three years, "a hale old man," who had enjoyed immunity from every trace of constitutional syphilis, although he had undergone infection as many as TWENTY-FOUR TIMES during his life time.—*Practitioner.*

---

A new Uterine Sound.—Dr. Murray presented to the Obstetrical Society of London, May 3, 1876, a flexible vertebrated sound, which could be introduced limp, and it can thus follow any irregularities of the uterine canal. After introduction it can be made firm in its course by means of a screw in the handle. Adhesions and extreme flexions can thus be detected.—*Ex.*

---

Inhalation of Ether.—D. C. writes as follows: Upon suggestion of trial, by an undergraduate of Chicago Medical College, W. H. Smith, I have found a great help to one of the many inconveniences of inhaling ether. In order to prevent the nausea which often follows the anæsthesia, the patient, besides avoiding eating for some hours before inhalation, should take from one to two ounces of brandy just before commencing the process. Suggestion may be of benefit to other country practitioners.

In some parts of China, doctors are paid a salary per family, so long as the members of it are in health; when sick the doctor's pay is stopped. Think of American Homœopathists being paid in that way!!

---

Dr. E. H. Pratt, of Wheaton, Ill., recently reported to a Homœopathic Medical Society the case of "a mother who had given birth to a child with a small Homœopathic pill in its throat, which had been occasioned by another child having swallowed a number of little pills, which frightened the mother during pregnancy!"

---

Consanguineous Marriages.—At the Deaf and Dumb School, at Barcelona, Spain, there have been admitted two hundred and fifty-three children, during thirty-one years; of these only fifteen were the issue of consanguineous parents. This constitutes very small ground for the belief of the danger of such alleged misalliances.—*Independencia Medica*, April, 1876.

---

The Homœopathic Question.—The committee of nine, to whom was referred the question of Homœopathy in the University, reported, the chairman, Dr. Foster Pratt, reading the report. The committee first presented a history of the controversy in the University over the question of Homœopathy and its introduction out of the litigations in the Supreme Court touching the question. The majority of the committee, Messrs. Pratt, Johnson, Andrews, Jerome, Chittock, Brown and Baker, recommended the adoption of the following resolutions:

*Resolved*, That we are not content with the existing situation of the medical department of the University, because in our opinion it is not calculated to maintain or advance medicine as a science, nor is it consistent with the honor or interest of the profession.

*Resolved*, That a State under our form of government cannot successfully teach either medicine or theology, and that the medical profession ought to be its own teacher and the guardian of its own honor.

*Resolved*, That we regard all legislative interference with the government of the University as unconstitutional, wrong in principle and harmful in its results.

*Resolved*, That section 4 of the constitution of this State Society be amended so as to read as follows, viz.: "Section 4. The resident members should be elected by vote of a majority present at any regular meeting, their eligibility having previously been reported upon by the Committee on Admission: *Provided*, That no person shall be admitted to membership who practices or professes to practice in accordance with any so-called 'party' or sectarian school of medicine, or who has recently graduated from a medical school whose professors teach or assist in teaching those who propose to graduate in or practice irregular medicine."

The resolutions are favored by the minority, Drs. Cutler and Hamilton, except the last, which they opposed.

The submission of the report was followed by an animated debate, Drs. Frothingham, Rynd and McLean opposing the resolutions, and Drs. Pratt and Post upholding them.

The first three resolutions were finally adopted by a vote of about sixty to thirty. The fourth resolution was laid over for one year, this being the usual course with amendments to the constitution. Prof. Frothingham thereupon announced that he would no longer stay in the society. The resolution changing the constitution was ordered to be sent to the local societies, and their views were asked for the guidance of the next convention.—*Daily Press.*

---

### STEVENS' TRIENNIAL PRIZE—1879.

This prize, established by Alexander H. Stevens, M. D., amounts to two hundred dollars. The questions for 1879 are as follows:

I. Bacteria, and kindred organisms in their relation to disease.

II. Human Excreta, as a cause of disease; the diseases produced therefrom, and their prevention by hygienic means.

Competing essays on either of the above subjects are expected to give an account of our present knowledge, and also the results of personal investigation. The essays must be sent in to the President of the College of Physicians and Surgeons, New York, on or before the first day of January, 1879. Each essay must be designated by a device or motto, and must be accompanied by a sealed envelope, bearing the same device or motto, and containing the name and address of the author. The envelope belonging to the successful essay will be opened, and the name of the author announced at the annual commencement of the College, in March, 1879.

This prize is open for universal competition.

J. C. DALTON, M.D.,
*Secretary of the Committee.*

---

MITCHELL, IND., Sept. 26, 1876.

The Second Annual Meeting of the Indiana, Illinois and Kentucky Tri-State Medical Society will be held in the city of Vincennes, Ind., Nov. 21, 1876. Prof. Byford, of Chicago, will deliver an address. G. W. BURTON, *Rec. Sec'y.*

---

Professor Leidesdorf, of Vienna, who was summoned to Constantinople to be consulted about the Sultan's disease, received the modest fee of £3,000 for his consultation.

---

The medical faculty of the University at Heidelberg, Germany, has recently sustained the loss of two members who stood in the foremost rank of the German surgeons. On Aug. 17, Prof. Von Chelius died at the age of eighty-three years. During his long and brilliant career he had attained a world-wide reputation as a teacher, diagnostician and operating surgeon. On Aug. 28, Heidelberg and the medical profession were called to mourn the death of another surgeon, equally eminent for his achievements in advancing operative surgery—Prof. Gustave Simon, then only fifty-two years of age, died of pulmonary phthisis. He was a most amiable, kind-hearted man; he was not a brilliant lecturer, but a splendid teacher, a patient and keen observer, a bold, though not reckless operator, and the master in the operation for vesico-vaginal fistula.

---

We have received the annual announcement of the College of Medicine of the Syracuse University, and notice that the revised course of instruction of the Boston school is closely copied in this young institution. There are the three courses of instruction, each extending from the first of October to the last of June, and divided into two terms; the studies graduated to the three courses of instruction; the instruction given by lectures and recitations; the examinations from time to time during the college study, and the assurance of preliminary English education by the requirement of either an academical degree or an examination as a condition to matriculation. The final examination is made by a board of censors who are not members of the faculty. When Harvard adopts this commendable feature she will be obliged to borrow from her youthful neighbor.

---

General Cialdini, the unpretending medical student who, in the year 1832, occupied wretched quarters in the Rue de la Harpe, and there translated into Italian the works of Velpeau and other authors, is about to revisit Paris in the capacity of Italian Minister.—*Gaz. Hebd.*

